# Supplementary material for: Process Design of Continuous Powder Blending Using Residence Time Distribution and Feeding Models
Source: Pharmaceutics. 2020 Nov 20;12(11):1119. doi: 10.3390/pharmaceutics12111119 (PMC7699818; doi:10.3390/pharmaceutics12111119)
Supplement: Supplementary file 1 [file pharmaceutics-12-01119-s001.pdf]

pharmaceutics

Supplementary Materials: Process design of continuous powder blending using residence time distribution and feeding models

Martin Gyürkés, Lajos Madarász, Ákos Köte, András Domokos, Dániel Mészáros, Áron Kristóf Beke, Brigitta Nagy, György Marosi, Hajnalka Pataki, Zsombor Kristóf Nagy and Attila Farkas

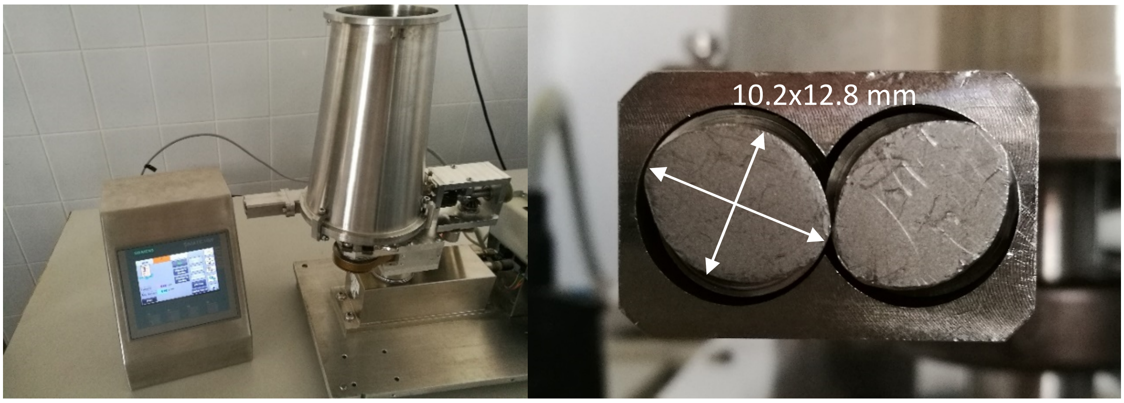

Figure S1. MechaCAD twin-screw feeder.

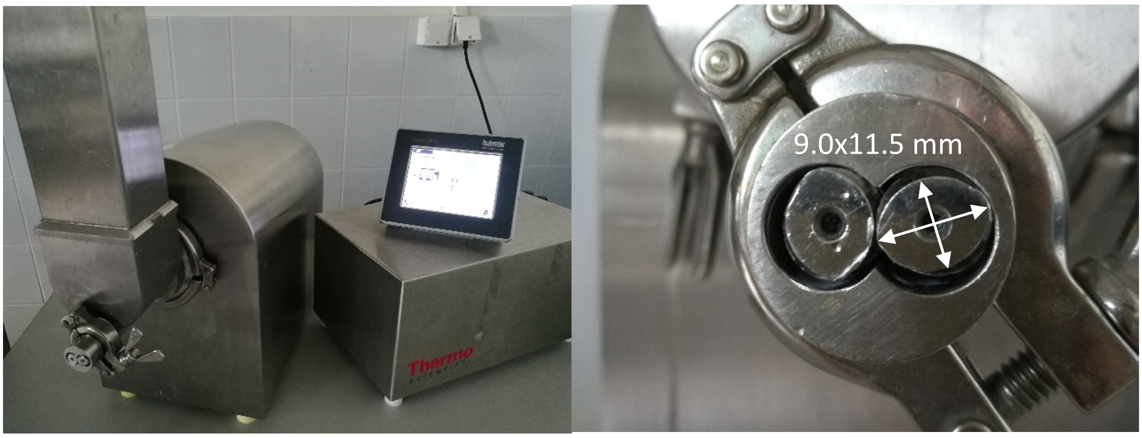

Figure S2. Brabender twin-screw feeder.

Table S1. Results of scale-up experiments carried out by changing mass flow rate from lab-scale 0.3 kg/h to pilot-scale 1 kg/h and constant 80 rpm screw rotational speed.

Mass flow [kg/h]	Screw Rotational Speed [rpm]	MRT [s]	σ [s]	$m_{hold-up}$ [g]
0.30	80	63.89	27.25	5.33
0.40	80	59.72	17.01	6.64
0.60	80	63.45	18.55	10.58
0.80	80	58.34	22.47	12.97
1.00	80	62.74	13.48	17.44

Table S2. Results of scaled-up experiments carried out by changing screw rotational speed from 40 rpm to 100 rpm and constant 0.3 kg/h mass flow.

Mass flow [kg/h]	Screw Rotational Speed [rpm]	MRT [s]	σ [s]	$m_{hold-up}$ [g]
0.30	100	55.93	21.89	4.66
0.30	80	63.89	27.25	5.33
0.30	60	70.63	20.36	5.89
0.30	40	99.03	22.55	8.26

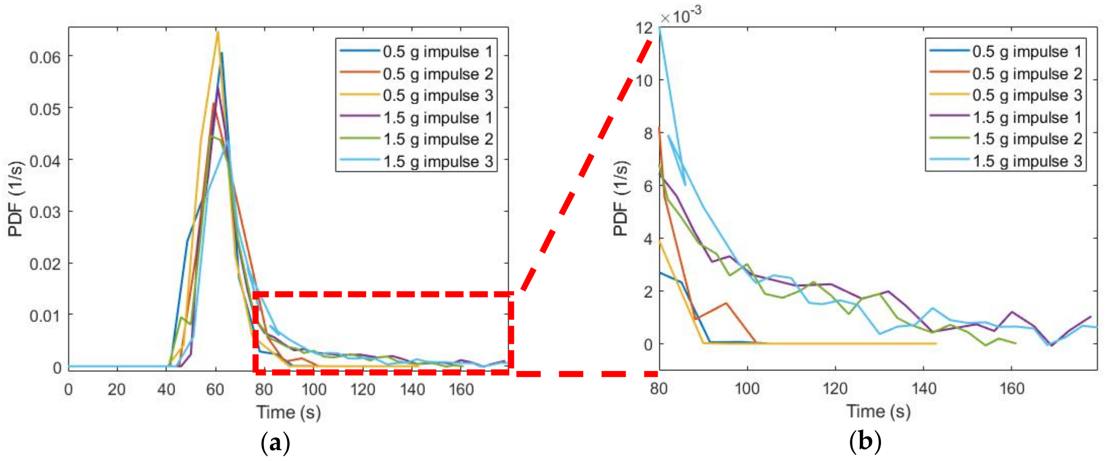

Figure S3. Impulse responses of 0.5 g and 1.5 g ASA disturbances on 0.30 kg/h mass flow and 60 RPM screw rotational speed setup: (**a**) Impulse response; (**b**) Impulse response zoomed to the tail of the distribution (t > 80 s).

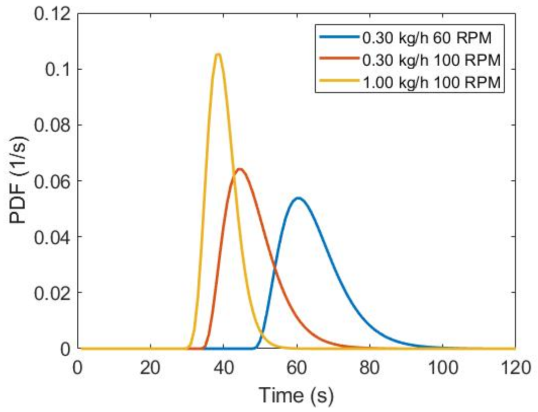

Figure S4. Effect of CPPs on the PDF. Screw rotational speed decrease dead time, and mass flow slightly decrease width.

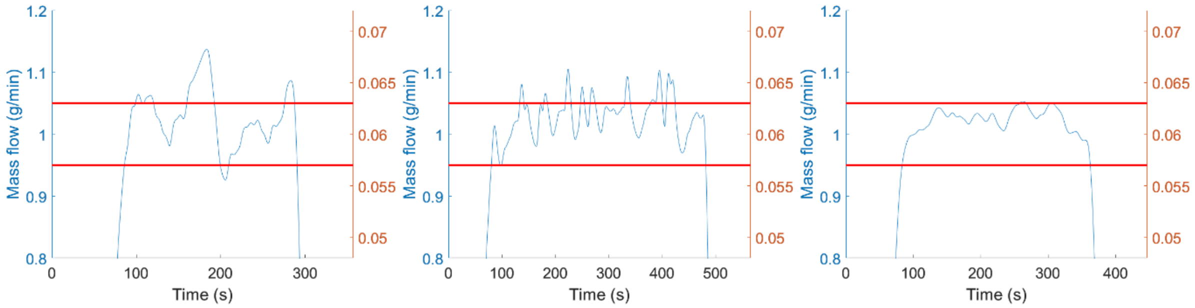

Figure S5. ASA mass flow after the powder blender based on lab-scale RTD model and the feeder characterization experiments: (**a**) MechaCAD; (**b**) Brabender; (**c**) Single-screw.
